# Prognosis after Palliative Surgery for Patients with Spinal Metastasis: Comparison of Predicted and Actual Survival

**DOI:** 10.3390/cancers14163868

**Published:** 2022-08-10

**Authors:** Hideaki Nakajima, Shuji Watanabe, Kazuya Honjoh, Yuya Izubuchi, Yumiko Watanabe, Takaaki Tanaka, Akihiko Matsumine

**Affiliations:** Department of Orthopaedics and Rehabilitation Medicine, Faculty of Medical Sciences, University of Fukui, 23-3 Matsuoka Shimoaizuki, Eiheiji-cho, Yoshida-gun, Fukui 910-1193, Japan

**Keywords:** spinal metastasis, palliative surgery, prognostic factors, revised Tokuhashi score, new Katagiri score, CRP/albumin ratio, adjuvant therapy

## Abstract

**Simple Summary:**

Increased options for cancer treatment have made the prediction of prognosis an important factor in therapeutic decision making. The aim of this study was to assess the clinical significance of prognosis–scoring systems and to identify predictors for 6–month mortality after palliative surgery. The median actual survival period was longer than the predicted life expectancy based on the revised Tokuhashi score and new Katagiri score. However, 21.3% of patients died of cancers within 6 months after palliative surgery. A statistical analysis showed that a higher CRP/albumin ratio (odds ratio: 0.39; cut–off 0.409) and absence of postoperative adjuvant therapy (odds ratio: 7.15) were independent risk factors for poor survival. Our findings suggest the need for careful consideration to determine if palliative surgery is the best option for a patient with these negative prognostic factors, regardless of life expectancy predicted based on a prognosis score.

**Abstract:**

Prediction of prognosis is a key factor in therapeutic decision making due to recent the development of therapeutic options for spinal metastases. The aim of the study was to examine predictive scoring systems and identify prognostic factors for 6–month mortality after palliative surgery. The participants were 75 patients with spinal metastases who underwent palliative surgery and had a minimum follow–up period of 1 year. Associations of actual survival with categories based on the revised Tokuhashi score and new Katagiri score were evaluated. Univariate and multivariate analyses were performed to identify prognostic factors for 6–month mortality after palliative surgery. The median actual survival period was longer than those predicted using the scoring systems. However, 21.3% of patients died of cancers within 6 months after surgery. A higher CRP/albumin ratio (odds ratio: 0.39; cut–off 0.409) and absence of postoperative adjuvant therapy (odds ratio: 7.15) were independent risk factors for 6–month mortality. There was no association of mortality with primary site, severity of sarcopenia, or other biomarkers. These results suggest that careful consideration is needed to determine whether palliative surgery is the best option for patients with a high preoperative CRP/albumin ratio and/or absence of postoperative adjuvant therapy, regardless of predictions made from scoring systems.

## 1. Introduction

The number of patients with cancer and deaths due to cancer have increased worldwide. Statistics from the International Agency for Research on Cancer, an external research organization of the World Health Organization, indicated 19.3 million new cases of cancer and estimated 10.0 million cancer deaths worldwide in 2020, a significant increase from the 2002 estimates of 10.9 million new cases and 6.7 million cancer deaths, respectively [1,2]. A previous study suggested that approximately 30% of patients with advanced cancer develop spinal metastasis [3]. In a cadaver study, spinal metastases were found in 36% of 832 deceased patients with a terminal diagnosis of malignant neoplasm [4]. Furthermore, 5–20% of these patients suffered from metastatic spinal cord compression (MSCC) [5,6]. Spinal metastasis can cause a number of sequelae, including pain, instability, and neurological deficit. In addition, patients with MSCC may have progression of myelopathy that results in the loss of motor, sensory, and autonomic functions.

Many reports have suggested the superiority of palliative surgery over nonsurgical treatment for the improvement in performance status (PS), activities of daily living (ADL), and neurological status [7,8]. The cost–utility of surgical treatment for spinal metastasis is also considered to be acceptable, especially for patients who are nonambulatory due to acute neurologic compromise [9,10]. With recent developments in therapeutic options for spinal metastases, including surgery, radiotherapy, chemotherapy, and targeted therapy, spine surgeons and oncologists need to consider the best option for remission of symptoms based on the prediction of life expectancy and the benefits of treatment.

At our hospital, patients with progressive neurological deficits or intractable pain were selected as candidates for palliative surgery if the life expectancy was predicted to be over approximately 6 months by using prognosis–scoring systems, opinions from oncologists, and the neurologic, oncologic, mechanical, and systemic (NMOS) framework [11]. However, contrary to our preoperative predictions, some patients survived for ≤6 months after palliative surgery. Thus, the aim of the study was to evaluate whether there is a discrepancy between prognosis prediction scores and actual prognosis, and to identify predictors of poor survival of ≤6 months after surgery.

## 2. Materials and Methods

### 2.1. Study Design

Between 2005 and 2021, 83 patients with spinal metastases underwent palliative surgery at our hospital. The study cohort included 75 patients who had a minimum follow–up period of 1 year, including those who died within 1 year after surgery (follow–up rate: 90.4%). The surgical indication was progressive neurological deficits due to MSCC and/or intractable pain associated with spinal metastases. Patients in poor general condition for general anesthesia and those with short life expectancy (<3–6 months) assessed by using prognosis–scoring systems and the opinions of oncologists were excluded from surgery. All patients underwent one–stage posterior decompression with or without fusion with reference to the spinal instability neoplastic score (SINS). To maintain case homogeneity, patients who received total en bloc spondylectomy as curative treatment were excluded. The study protocol was approved by the Human Ethics Review Committee of Fukui University Medical Faculty and strictly followed the Clinical Research Guidelines of the Ministry of Health, Labor, and Welfare of the Japanese Government.

### 2.2. Clinical Assessments

Data were collected on patient background (age, sex, and body mass index (BMI)), primary site, preoperative Frankel grade [12], Eastern Cooperative Oncology Group (ECOG) PS [13] at 3 months after surgery assessed by oncologists, affected level, indication for surgery (neurological deficit, intractable pain), surgical procedure (decompression and fusion, decompression without fusion), surgery–related factors (operation time, blood loss, emergency operation, perioperative complications of Clavien–Dindo classification grade 2 or higher [14]), and pre– and postoperative adjuvant therapy (radiation, chemotherapy, hormonal therapy, and molecular targeted drugs). Both the revised Tokuhashi score [15] and new Katagiri score [16] were used for the prediction of prognosis and assessment of the severity of spinal metastases. SINS was used to assess the degree of spinal instability and to guide the need for fusion surgery. Sarcopenia was evaluated using the sum of the left and right psoas area on an axial image in the mid–L3 vertebral body divided by the L3 vertebral body area, calculated using a picture archiving and communication system [17]. Radiological assessments were performed by two observers. The preoperative inflammatory response and nutritional status were evaluated using the serum C–reactive protein (CRP)/albumin ratio (CAR), neutrophil/lymphocyte ratio (NLR), and platelet/lymphocyte ratio (PLR) [18]. The postoperative survival period was defined as the time from the date of surgery to the final follow–up or death, whichever was earlier. Information on death was obtained from the patient’s family or from a transfer hospital.

### 2.3. Statistical Analysis

Data were presented as a median [interquartile range]. Categorical variables were compared by Mann–Whitney U–test or chi–square test, with *p* < 0.05 considered to be significant. Significant factors in univariate analysis and those reported in the literature were included in a multivariate regression model. The estimated odds ratio and 95% confidence interval (CI) were calculated to identify independent predictors of survival >6 months. For measurement of radiological parameters, inter– and intraobserver reliabilities were assessed by calculating intraclass correlation coefficients (ICCs), with ICC (1,3) and ICC (2,3) > 0.75 considered to represent good to excellent reliability. The median survival period based on the predictive scoring systems were calculated using Kaplan–Meier survivorship analysis. The cut–off was defined as the point nearest to the upper–left corner of a receiver operating characteristic (ROC) curve, and the area under the curve (AUC) was used to assess the accuracy of the parameter as a predictor. EZR (Saitama Medical Center, Jichi Medical University, Saitama, Japan) [19], a graphical user interface for R (The R foundation for Statistical Computing, Vienna, Austria), was used for all analyses.

## 3. Results

### 3.1. Comparison of Preoperative Prediction and Actual Median Survival Time after Palliative Surgery

**Figure 1:**
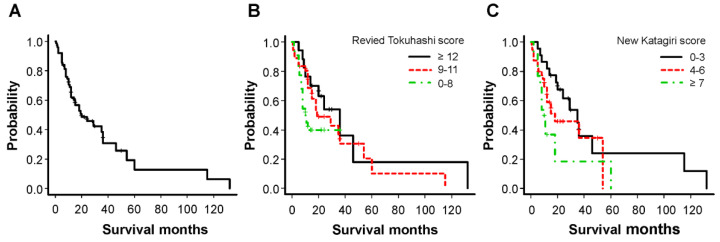
Kaplan−Meier analyses of overall survival after palliative surgery in all patients (**A**) and categories based on the revised Tokuhashi score (**B**) and new Katagiri score (**C**).

A total of 75 patients (median age: 67.0 years; male 53, female 22) were enrolled in the study. The primary site and median survival period are shown in Table 1. There was no significant difference in survival based on the primary site. The overall Kaplan–Meier median survival for all patients was 20 months (12–36) (Figure 1A). Kaplan–Meier survival curves based on the revised Tokuhashi score (good, ≥12; intermediate, 9–11; poor, 0–8) and new Katagiri score (good, 0–3; intermediate, 4–6; poor, ≥7) for the prognosis are shown in Figure 1B,C, respectively. The median survival periods could be clustered using these scoring systems: 36.0, 19.0, and 10.5 months using the revised Tokuhashi score (*p* = 0.13 by log rank test) and 35.0, 18.0, and 10.0 months using the new Katagiri score (*p* = 0.055 by log rank test). The 1–year survival rates in the poor and intermediate groups were 77.8% and 38.1% using the revised Tokuhashi score and 30.8% and 65.0% using the new Katagiri score, respectively. Of the 75 patients, 16 (21.3%) died of various cancers within 6 months of surgery, contrary to our preoperative predictions. Table 2 shows the relationship between the revised Tokuhashi score and the new Katagiri score with 6–month mortality. In some cases, a discrepancy between these predictive scoring systems and the actual survival was observed; even patients clustered based on intermediate and good groups died (19.4%/22.5% and 5.9%/9.1% of patients, respectively) within 6 months of surgery. There was no correlation between the median survival period and SINS: 14, 35 and 18 months in stable (0–6 points), potentially unstable (7–12 points), and unstable (≥13 points) cases, respectively (*p* = 0.38 by log rank test) were observed.

### 3.2. Prognostic Factors for Poor Survival of Less than 6 Months after Palliative Surgery

**Figure 2:**
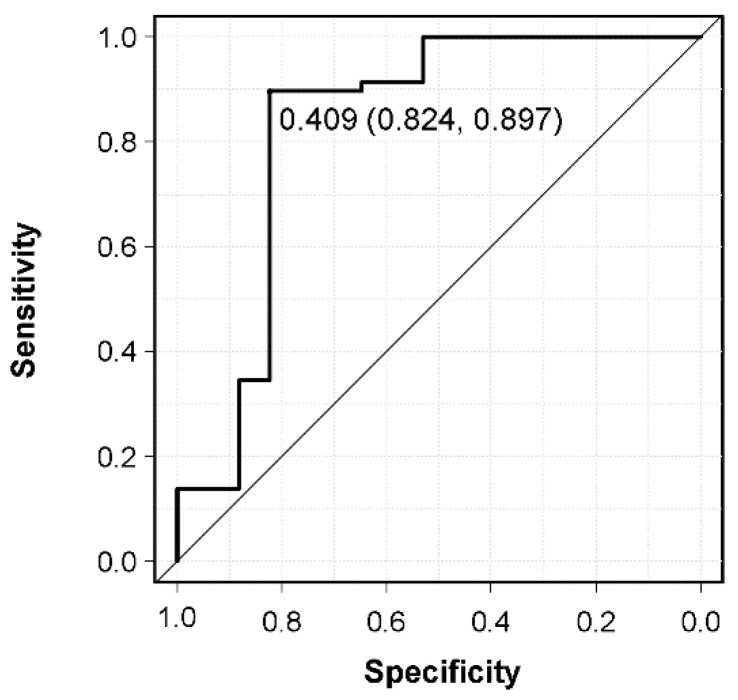
Receiver operating characteristic curve of preoperative CRP/albumin ratio (CAR) for 6–month mortality after palliative surgery and determination of the CAR cut–off value.

A comparison of patients with and without survival >6 months is shown in Table 3. In univariate analysis, preoperative Frankel grade, CAR, and postoperative adjuvant therapy differed significantly between these groups. However, in terms of preoperative Frankel grade, there was no significant difference in the survival period between nonambulatory (Frankel A–C) and ambulatory (Frankel D and E) cases (*p* = 0.56). The median survival of 6 patients with a survival period of ≤6 months without any postoperative adjuvant therapy was 1.75 [1.5–2.0] months, which was a very poor survival rate. In three of these six patients, the primary site was first detected owing to spinal metastasis, and no pathological diagnosis was performed preoperatively (lung cancer without molecularly targeted drugs (n = 2), hormone–independent prostate cancer (n = 1)). The remaining three cases showed rapidly progressing cancer (colorectal cancer, gastric cancer, and neck cancer); unfortunately, they could not achieve the expected physical performance postoperatively. There was no difference in the prognostic scores (revised Tokuhashi score, new Katagiri score) between the two groups, or in patient background, primary site, postoperative ECOG PS, affected level, severity of sarcopenia, NLR, PLR, indication for surgery, surgical procedures, operation factors, and preoperative adjuvant therapy. Of the 75 patients, 11 (14.7%) experienced perioperative complications within 14 days of surgery (surgical site infection (n = 3), motor weakness (n = 3), pneumonia (n = 2), severe anemia (n = 2), and deep vein thrombosis (n = 1)), which were not responsible for their mortality. Multivariate logistic regression analysis including significant variables from univariate analysis and prognostic scores was used to identify independent prognostic factors in patients with the survival of >6 months. In this analysis, CAR (odds ratio 0.39) and postoperative adjuvant therapy (odds ratio 7.15) were identified as independent prognostic factors (Table 4). In addition, prediction of the preoperative CAR for 6–month mortality was calculated using an ROC curve. The AUC for preoperative CAR was 0.832, and the cut–off value for 6–month mortality after palliative surgery derived from the ROC curve was 0.409 (sensitivity, 89.7%; specificity, 82.4%) (Figure 2).

### 3.3. Association between Prognostic Scoring Systems and CRP/Albumin Ratio (CAR)

In the multivariate analysis, a higher CRP/albumin ratio (cut–off: 0.409) was found to be an independent risk factor for 6–month mortality. Next, the relationships between prognostic–scoring systems and preoperative CAR were assessed (Table 5). For the revised Tokuhashi score, the preoperative CAR in poor survival cases (score 0–8) was significantly higher than in those with predicted intermediate (score 9–11) and good (score ≥ 12) survival (*p* = 0.024). However, the median preoperative CAR (0.37) was below the cut–off value (0.409). There was no association between the new Katagiri score for prognosis and the preoperative CAR (*p* = 0.22).

## 4. Discussion

Palliative surgery for patients with spinal metastases is likely to increase owing to advances in cancer treatment that have increased the life expectancy for primary tumors, including molecular targeted drugs for lung and renal cancers and hormonal therapy for breast and prostate cancers. Predicting the prognosis for patients with spinal metastases is an important factor in therapeutic decision making. Candidates for palliative surgery are identified using prognostic scores and opinions of oncologists; however, 21.3% of the patients in the current study had survival of ≤6 months after palliative surgery. Thus, we retrospectively examined the revised Tokuhashi and new Katagiri scores and identified the prognostic factors for poor survival in patients with the survival of ≤6 months after palliative surgery, contrary to preoperative assessments.

Prognostic scores are used to categorize patients with spinal metastases based on the survival time and are useful guides in therapeutic decision making [20,21,22]. In our patients, the revised Tokuhashi and new Katagiri scores were effective for categorizing their prognosis into poor, intermediate, and good survival, although without statistical significance. The original article on the revised Tokuhashi score found life expectancies of patients in the poor (score 0–8) and intermediate (score 9–11) groups of <6 and >6 months, respectively [15]. However, the respective median survival periods in the current study were 10.5 and 18 months for these groups, which differed from the original study. In contrast, 19.4% and 5.9% of patients categorized in the intermediate and good survival groups, respectively, based on the revised Tokuhashi score died within 6 months of surgery. Previous studies have suggested that the agreement of the actual survival with that predicted using the revised Tokuhashi score is approximately 60%, and that the predictive accuracy decreases for patients with a life expectancy shorter than 1 year [23,24,25]. The new Katagiri score has advantages of including classification of the growth rate of the primary cancer and effects of molecular targeted drugs and sensitivity to hormonal therapy. The original study on the new Katagiri score reported 1–year survival rates of 6% in the poor group (score ≥ 7) and 49% in the intermediate group (score 4–6) [16]. However, the 1–year survival rates in the current study were 30.8% and 65.0% in these respective groups, showing a similar discrepancy from the original study to that for the revised Tokuhashi score. Another study on the survival rate after palliative surgery using the new Katagiri score found a rate of 22% in the poor group [26]. The Tokuhashi score was published in 2005 and the Katagiri score was applied to patients between 2005 and 2008; this is one of the shortcomings of using fixed algorithmic scoring systems. Our results suggest that recent developments in cancer treatment have prolonged the life expectancy. On the other hand, 22.5% and 9.1% of patients categorized in the intermediate and good survival groups based on the new Katagiri score died within 6 months of surgery. Regarding SINS, the association with the prognosis for survival is controversial [27,28]. The current study did not show any association of SINS or surgical procedures with the survival period. SINS is an important scoring system to assess spinal instability and determine the need for fusion surgery [29]; however, it may not predict the survival after palliative surgery based on the results of the current study.

Preoperative ambulatory status has been identified as a significant prognostic factor in patients with spinal metastases in a meta–analysis, with the suggestion that this status should be considered when choosing the treatment modality [30]. In the current study, the preoperative Frankel grade differed significantly between patients with and without survival for >6 months in univariate analysis; however, no significant difference was observed in the survival period between nonambulatory (Frankel A–C) and ambulatory (Frankel D and E) cases. Frailty and/or elderly age may also increase the risk of mortality, postoperative complications and chemotherapy intolerance [31,32]. A cohort study suggested that the psoas size, as a surrogate for frailty/sarcopenia, predicts the overall mortality more strongly than the Tokuhashi score [33]. However, in the current study, the survival period was not correlated with age, BMI, or psoas size at the L3 vertebra body. A modified frailty index used in this study may not capture the systemic condition and functional status of the patients. A previous article suggested that a frailty measure specific to spinal metastatic disease should be developed for highlighting systemic variables and patient factors that affect clinical outcomes [17].

Inflammatory mediators and cytokines produced by inflammatory cells in the tumor microenvironment are increasingly recognized as important contributors to cancer progression through their effects on the promotion of proliferation, angiogenesis, and metastasis, reduction in responses to hormones and chemotherapeutic agents, and subversion of adaptive immunity [34]. The association between inflammatory response and tumor progression also suggests the possible prognostic significance of preoperative inflammatory biomarkers, including CAR, NLR, and PLR. Several cancer studies have suggested the accuracy of CAR. One of the studies even reported CAR to be the most sensitive prognostic factor for esophageal squamous cell carcinoma [35]. In the current study, CAR was an independent prognostic factor for the survival period of over 6 months, and the cut–off for CAR was 0.409. In another study on patients with spinal metastasis from clear cell renal cell carcinoma, NLR and PLR were found to be significantly correlated with the overall survival [18]. Further studies are needed to identify the optimal biomarker for each primary site; however, preoperative CAR may be a reliable inflammatory biomarker to predict life expectancy. Importantly, the findings of the current study also indicated that CAR was not clearly associated with the revised Tokuhashi and new Katagiri scores. Thus, careful consideration should be given to whether palliative surgery is the best option for a patient with a high CAR, especially in a case with relatively good prognosis scores.

The prolonged prognosis of cancer patients is largely due to recent developments in chemotherapy, hormonal therapy and molecular targeted therapy; however, not all patients are eligible for these therapies. In the original study of the new Katagiri score, previous chemotherapy was a negative prognostic factor for survival rate (odds ratio: 1.39) [16]. In the current study, a history of preoperative adjuvant therapy was not associated with a poor survival period; however, the absence of a postoperative adjuvant therapy was identified as an independent negative factor for 6–month mortality after palliative surgery. Importantly, the hazard ratio differed significantly between treatment with molecular targeted drugs or hormonal therapy and between sensitive and resistant lesions, as well as sensitivity to chemotherapy and radiotherapy, even for the same primary cancer [26]. Thus, preoperative information from oncologists on the indication for postoperative adjuvant therapy is particularly important when determining the prognosis for a primary cancer, especially for considering the indication for palliative surgery. In cases of spinal metastases with motor paralysis and/or intractable pain, preoperative PS does not necessarily reflect the general condition. Therefore, oncologists and spine surgeons should use decision making frameworks such as NOMS in their decision making [11]. Nevertheless, in cases of spinal metastases that require urgent palliative surgery without any preoperative detailed pathological diagnosis because of spinal metastasis, the informed consent about the prognosis should be obtained more carefully.

This study has certain limitations, including its retrospective and single-center design, small number of patients, and inclusion of cases receiving surgical treatment only. The number of cases for each primary site might be too small for identification of prognostic factors and treatment strategies for each site. Thus, larger prospective and multicenter clinical studies are needed to provide further evidence in support of our findings. Despite these limitations, the results of the study provide important insights and guidance on therapeutic decision making for patients with spinal metastases.

## 5. Conclusions

Recent developments in cancer treatment and the variety of treatment options have made the prediction of prognosis an important factor in therapeutic decision making. The revised Tokuhashi score and the new Katagiri score are useful for predicting the prognosis of patients with spinal metastasis; however, the median life expectancy in the current study was longer than the survival period predicted using these scoring systems. Contrary to the preoperative predictions, 21.3% of patients had a survival period of <6 months after palliative surgery. A higher CRP/albumin ratio (CAR) and absence of postoperative adjuvant therapy were independent risk factors for 6–month mortality, regardless of preoperative life expectancy based on scoring systems and/or the opinions of oncologists. Our findings suggest that patients with more favorable prognostic scores, but with high CAR (cut–off value = 0.409) and/or lack of eligibility for postoperative adjuvant therapy, may have shorter survival than expected and that careful consideration should be given to determine whether palliative surgery is the best option for such patients.

## Figures and Tables

**Table 1:** Primary site and median survival times in patients with spinal metastasis.

Primary Site	Number of Patients	Median Survival Time	*p* Value
Total	75	20	
Prostate cancer	21	36	0.25
Renal cell carcinoma	18	18	
Lung cancer	12	14	
Colon cancer	9	60	
Breast cancer	6	41	
Others	9	12	

**Table 2:** Relationship between the predictive prognostic scores with 6–month mortality.

Scoring System	Poor	Intermediate	Good	*p* Value
Revised Tokuhashi score, n (%)	8/22 (36.4%)	7/36 (19.4%)	1/17 (5.9%)	0.065
New Katagiri score, n (%)	5/13 (38.5%)	9/40 (22.5%)	2/22 (9.1%)	0.12

Data are presented as the number of patients died within 6 months of surgery/total number of patients (%).

**Table 3:** Comparison of patients with and without survival >6 months after palliative surgery.

Item	Survival ≤ 6 Months	Survival > 6 Months	*p* Value
**Patients**, n (%)	16 (21.3%)	59 (78.7%)	
**Patient background**			
Age (years), median [IQR]	68.0 [62.5, 73.0]	66.5 [60.0, 73.3]	0.86
Sex (Male, n (%)/Female, n (%))	13 (81.3%)/3 (18.8%)	40 (67.8%)/19 (32.2%)	0.46
BMI (kg/cm^2^), median [IQR]	22.2 [19.8, 23.5]	20.9 [17.7, 22.2]	0.23
**Primary site,** n (%)			
Prostate	5 (31.3%)	16 (27.1%)	0.87
Kidney	4 (25.0%)	14 (23.7%)	
Lung	3 (18.8%)	9 (15.3%)	
Colon	2 (12.5%)	7 (11.9%)	
Breast	0 (0 %)	6 (10.2%)	
Others	2 (12.5%)	7 (11.9%)	
**Preoperative Frankel grade**, n (%)			
A	1 (6.3%)	3 (5.1%)	0.0055 *
B	3 (18.8%)	2 (3.4%)	
C	3 (18.8%)	28 (47.5%)	
D	4 (25.0%)	2 (3.4%)	
E	5 (31.3%)	24 (40.7%)	
**PS at 3 months post–op = 3 or 4**, n (%)	9 (56.3%)	21 (35.6%)	0.23
**Affected level**, n (%)			
Cervical	2 (12.5%)	6 (10.2%)	0.42
Thoracic	13 (81.3%)	41 (69.5%)	
Lumbar	1 (6.3%)	12 (20.3%)	
**L3 psoas/vertebra** (mm^2^), median [IQR]	0.82 [0.65, 1.15]	0.72 [0.61, 0.85]	0.37
**Scoring systems,** median [IQR]			
Revised Tokuhashi score	10.0 [7.5, 11.5]	9.0 [7.8, 11.0]	0.59
New Katagiri score	6.0 [5.0, 6.0]	4.0 [3.0, 6.0]	0.10
SINS	8.0 [5.0, 9.0]	9.0 [7.0, 12.0]	0.11
**Inflammatory biomarkers,** median [IQR]			
CRP/albumin ratio	0.82 [0.65, 1.4]	0.09 [0.02, 0.20]	0.032 *
Neutrophil/lymphocyte ratio	5.28 [3.32, 6.22]	3.25 [2.69, 5.02]	0.24
Platelet/lymphocyte ratio	191.8 [81.9, 321.9]	190.5 [101.2, 268.5]	0.91
**Indication for operation**, n (%)			
Paralysis	9 (56.3%)	32 (54.2%)	1
Intractable pain	7 (43.8%)	27 (45.8%)	
**Surgical procedure**, n (%)			
Decompression and fusion	13 (81.3%)	50 (84.7%)	1
Decompression without fusion	3 (18.8%)	9 (15.3%)	
**Surgery–related factors**			
Operation time, median [IQR]	190 [149.5, 341]	262.5 [215, 356.8]	0.36
Blood loss, median [IQR]	580 [200, 625]	640 [200, 1520]	0.243
Emergency operation, n (%)	9 (56.3%)	29 (49.2%)	0.83
Perioperative complications	3 (18.8%)	8 (13.6%)	0.49
**Adjuvant therapy,** n (%)			
Preoperative adjuvant therapy	9 (56.3%)	35 (59.3%)	1
Postoperative adjuvant therapy	10 (62.5%)	55 (93.2%)	0.0053 *

IQR: interquartile range; PS: performance status; SINS: spinal instability neoplastic score, * *p* < 0.05.

**Table 4:** Multivariate analysis of predictors of survival >6 months after palliative surgery.

Variables	OR	95% CI	*p* Value
CRP/albumin ratio (per ratio)	0.39	0.19–0.81	0.025 *
Postoperative adjuvant therapy (Yes vs. No)	7.15	1.18–43.4	0.033 *
New Katagiri score (per score)	0.61	0.36–1.04	0.07
Revised Tokuhashi score (per score)	1.17	0.90–1.53	0.12

OR: odds ratio; CI: confidence interval; CRP: C–reactive protein, * *p* < 0.05.

**Table 5:** Relationships of predictive prognostic scores with the preoperative CRP/albumin ratio.

Scoring System	Poor	Intermediate	Good	*p* Value
Revised Tokuhashi score	0.37 [0.09, 1.03]	0.08 [0.05, 0.20]	0.05 [0.01, 0.10]	0.024 *
New Katagiri score	0.08 [0.05, 0.21]	0.18 [0.05, 1.21]	0.08 [0.04, 0.10]	0.22

Data are shown as median [interquartile range], * *p* < 0.05.

## Data Availability

The data presented in this study are available on request from the corresponding author and subject to the ethical approvals in place and materials transfer agreements.
